# Effects of extended powered knee prosthesis stance time via visual feedback on gait symmetry of individuals with unilateral amputation: a preliminary study

**DOI:** 10.1186/s12984-019-0583-z

**Published:** 2019-09-11

**Authors:** Andrea Brandt, William Riddick, Jonathan Stallrich, Michael Lewek, He Helen Huang

**Affiliations:** 1 Joint Department of Biomedical Engineering, North Carolina State University, 4402D Engineering Building III, NC State University, Raleigh, NC 27606 USA; 2 The University of North Carolina at Chapel Hill, Chapel Hill, NC 27599 USA; 3 Mission Gait, Richmond, VA 23228 USA; 4 Department of Statistics, North Carolina State University, Raleigh, NC 27606 USA; 5 Department of Allied Health Sciences, Division of Physical Therapy, The University of North Carolina at Chapel Hill, Chapel Hill, NC 27599 USA

**Keywords:** Gait; Amputation; Visual Feedback; Rehabilitation; Knee Prosthesis

## Abstract

**Background:**

Establishing gait symmetry is a major aim of amputee rehabilitation and may be more attainable with powered prostheses. Though, based on previous work, we postulate that users transfer a previously-learned motor pattern across devices, limiting the functionality of more advanced prostheses. The objective of this study was to preliminarily investigate the effect of increased stance time via visual feedback on amputees’ gait symmetry using powered and passive knee prostheses.

**Methods:**

Five individuals with transfemoral amputation or knee disarticulation walked at their self-selected speed on a treadmill. Visual feedback was used to promote an increase in the amputated-limb stance time. Individuals were fit with a commercially-available powered prosthesis by a certified prosthetist and practiced walking during a prior visit. The same protocol was completed with a passive knee and powered knee prosthesis on separate days. We used repeated-measures, two-way ANOVA (alpha = 0.05) to test for significant effects of the feedback and device factors. Our main outcome measures were stance time asymmetry, peak anterior-posterior ground reaction forces, and peak anterior propulsion asymmetry.

**Results:**

Increasing the amputated-limb stance time via visual feedback significantly improved the stance time symmetry (*p* = 0.012) and peak propulsion symmetry (*p* = 0.036) of individuals walking with both prostheses. With the powered knee prosthesis, the highest feedback target elicited 36% improvement in stance time symmetry, 22% increase in prosthesis-side peak propulsion, and 47% improvement in peak propulsion symmetry compared to a no feedback condition. The changes with feedback were not different with the passive prosthesis, and the main effects of device/ prosthesis type were not statistically different. However, subject by device interactions were significant, indicating individuals did not respond consistently with each device (e.g. prosthesis-side propulsion remained comparable to or was greater with the powered versus passive prosthesis for different subjects). Overall, prosthesis-side peak propulsion averaged across conditions was 31% greater with the powered prosthesis and peak propulsion asymmetry improved by 48% with the powered prosthesis.

**Conclusions:**

Increasing prosthesis-side stance time via visual feedback favorably improved individuals’ temporal and propulsive symmetry. The powered prosthesis commonly enabled greater propulsion, but individuals adapted to each device with varying behavior, requiring further investigation.

## Introduction

Establishing gait symmetry is a major aim of amputee rehabilitation to reduce the risk of secondary complications that often accompany asymmetric gait (e.g. back pain, osteoarthritis) [1–4]. Individuals with transfemoral amputation commonly exhibit reduced stance time and ground reaction forces from the prosthetic limb compared to their intact limb [5–8], coinciding with increased prevalence of secondary complications relative to individuals with more distal levels of amputation [3, 4]. Reduced propulsion from one limb has been associated with increased energy cost of walking [9, 10]. Yet, prior work has suggested asymmetry is a functional result of the biomechanical constraints of energetically-passive prostheses that need not be corrected [11–13]. Of note, passive prostheses cannot provide the necessary levels of joint torque and power during walking. The advent and commercialization of powered prostheses capable of mimicking the force generating behavior around biological joints [14–16] provides reason to revisit this perspective on gait symmetry.

Powered knee prostheses reduce biomechanical constraints by partially replacing the function of lower-limb biological muscles. To match the user, however, powered prostheses need to be set specifically for each individual. This personalization of powered knee control parameters can influence users’ gait symmetry [17], and the ability of a powered prosthesis to provide net power leads us to believe these devices can reduce propulsive and temporal asymmetry; however, amputees typically spend less time on the powered prosthesis compared to the intact side, even with personalized control parameters [17–19]. Because amputees appear to maintain temporal walking strategies across devices [20], this asymmetric behavior may transfer to powered devices as well. We postulate that this transfer of a previously-learned maladaptive motor pattern limits the functionality of more advanced prostheses.

Previously, real-time visual feedback has induced immediate improvements in temporal, spatial, and force symmetry for amputees walking with passive prostheses [21], and a case report demonstrated kinematic and metabolic improvements with such training [22]. To our knowledge, no one has investigated the ability and associated effects of amputees walking with a *powered* knee prosthesis to modulate their gait in response to visual feedback. This knowledge may improve amputees’ gait asymmetries that persist in spite of technological advancements and broaden the number of amputees that are candidates for powered prostheses.

The objective of this study was to preliminarily investigate the effects of increasing stance time on a commercially-available powered knee prosthesis via visual feedback on amputees’ gait symmetry without a walking aid. The ability to attain and maintain complete symmetry may be unrealistic given the biomechanical constraints that persist with powered prostheses (e.g. limited ankle motion), so our paradigm simply encourages a greater level of symmetry via increased stance time on the prosthetic limb. We hypothesized that with visual feedback targeting longer stance times, amputees would walk with improved temporal and force symmetry and reduced prosthesis-side hip power due to increased propulsion from the prosthesis. Second, we hypothesized that temporal measures may be similar between devices due to the transfer of similar motor patterns, but the powered prosthesis would enable greater improvements in propulsion, hip power, and perceived difficulty, considering its functional advantages.

## Methods

### Participants and experimental setup

We initially screened 11 people and recruited five people to participate in this study. All participants reported daily prosthetic wear for 12 or more hours and experience with treadmill walking (Table 1). Participants were excluded if they exhibited body weight/limb clearance not meeting Power Knee™ (Össur; Reykjavik, Iceland) limitations and vision/cardiovascular complications that may affect his/her ability to complete the protocol. All subjects provided informed, written consent to participate in our protocol approved by the Institutional Review Board of the University of North Carolina at Chapel Hill.

**Table 1:** Participant information

Subject	1	2	3	4	5
Gender	Male	Male	Male	Male	Female
Height	1.8 m	1.8 m	1.7 m	1.8 m	1.7 m
Body Weight	69 kg	94 kg	61 kg	69 kg	49 kg
Age	24 years	59 years	27 years	19 years	52 years
Time since Amputation	7 years	47 years	4 years	19 years	27 years
Reason for Amputation	Cancer	Cancer	Trauma	Congenital	Trauma
Side of Amputation	Right transfemoral amputation	Left transfemoral amputation	Right knee disarticulation	Left transfemoral amputation	Right transfemoral amputation
Prescribed Prosthesis	Genium (Ottobock, 1.7 kg)	Genium (Ottobock, 1.7 kg)	Plie 3 (Freedom Innovations, 1.2 kg)	Rheo Knee (Ossur, 1.6 kg)	Power Knee (Ossur, 3.2 kg)
Time with Current Prosthesis	6 years	3 years	3 years	6 months	5 years
Physical Therapy Post-amputation	2 months	6 months	9 months	None	3 or more months
Suspension	Double-wall with pin to outer socket	Ischial containment suction	Shuttle lock, 3–5-ply socks, window socket with lanyard	Suction	Vacuum
Self-reported Functional K-level	3	3	3	4	3
Self-selected Walking Speed with Each Prosthesis	Prescribed: 0.8 m/s Powered: 0.8 m/s	Prescribed: 0.8 m/s Powered: 0.8 m/s	Prescribed: 0.9 m/s Powered: 0.7 m/s	Prescribed: 0.5 m/s Powered: 0.7 m/s	Powered (i.e. prescribed): 0.5 m/s

The listed prescribed prosthesis for Subjects 1–4 are energetically-passive knee prostheses. Subject 5 wore a powered prosthesis daily and was only tested with the powered prosthesis

The passive prostheses used in this study were Subjects 1–4’s prescribed, microprocessor-controlled, energetically-passive knee prostheses (Table 1). The commercially-available powered knee prosthesis used in this study was the Power Knee™ (Össur; Reykjavik, Iceland). Subject 5 used this powered prosthesis daily and did not have a passive prosthesis for comparison. A certified prosthetist, trained in alignment and fitting of the powered prosthesis, aligned and tuned the powered prosthesis for Subjects 1–4 (Table 2). Subject 5 used her daily settings. Due to his long residual limb, Subject 3 wore a low-profile high-performance foot similar to his prescribed foot, so that he did not need a lift on his intact foot to obtain proper alignment. Subject 5 wore her prescribed foot (Proflex; Össur; Reykjavik, Iceland)), and the remaining subjects wore the ProFlex XC (Össur; Reykjavik, Iceland) with the powered knee (for optimal performance of the powered knee) and their prescribed foot with their prescribed knee. Each subject maintained his/her same socket, suspension, and shoes across all testing conditions to reduce confounding effects.

**Table 2:** Commercially-available powered knee prosthesis settings

	Thigh length	Knee center height	Foot size	Pre-swing thigh angle	Maximum flexion angle	Swing initiation angle
1	48 cm	50 cm	26 cm	20	60	15
2	42 cm	55 cm	26 cm	30	60	10
3	54 cm	35 cm	26 cm	20	60	15
4	55 cm	46 cm	26 cm	28	60	20
5	43 cm	53 cm	26 cm	Unknown; set by her own prosthetist		

### Visual feedback

Due to the high variability in amputees’ spatial symmetry [23] and observed difficulty of inter-limb symmetry measures [21], we chose a unilateral, temporal metric (i.e. prosthetic-limb stance time) for visual feedback. More complex measures may also require longer training periods and the assistance of a physical therapist [24], limiting the accessibility of the intervention.

**Fig. 1:**
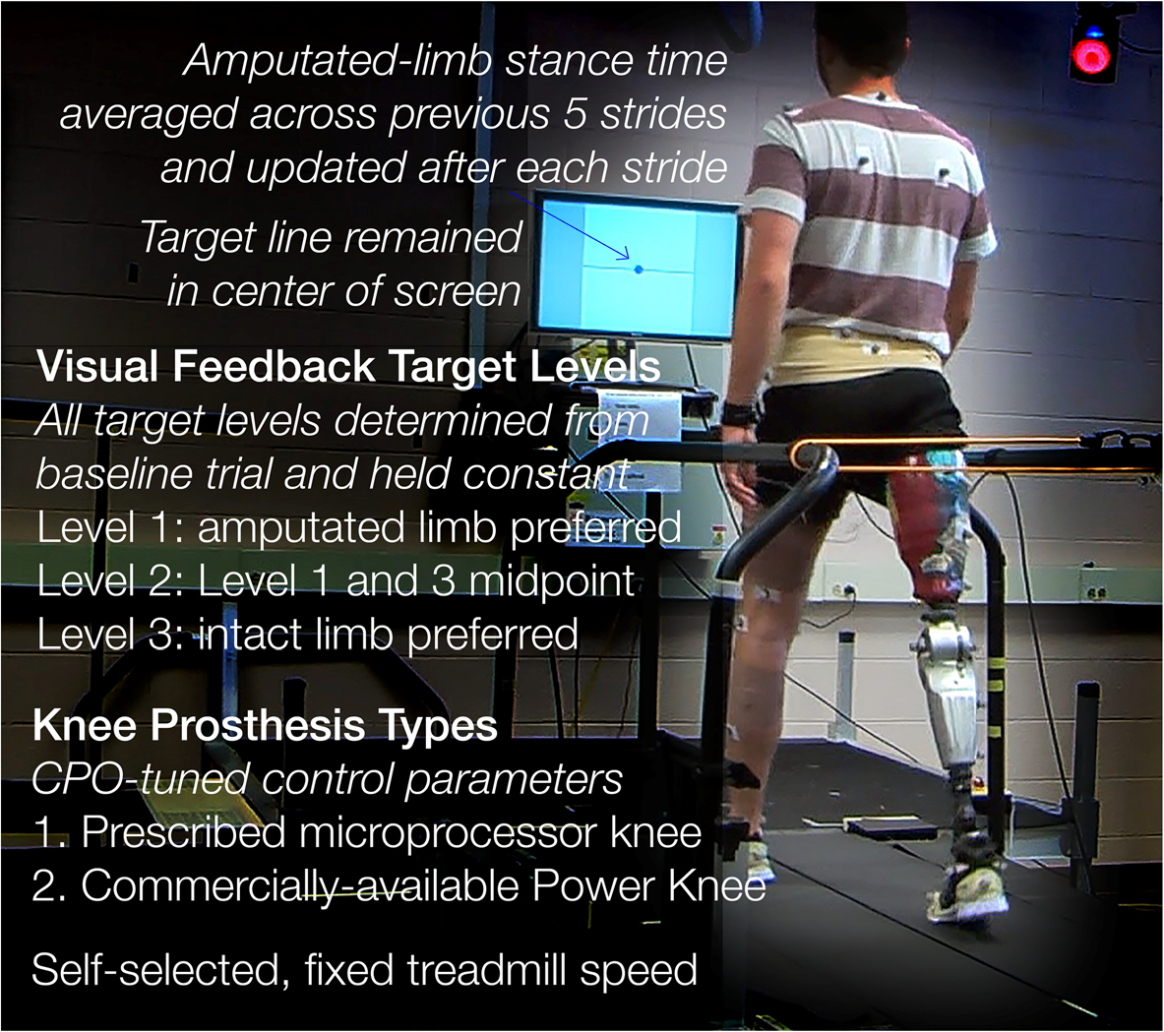
Experimental design and visual feedback display. We asked each subject to walk at their self-selected treadmill speed with their prescribed passive knee prosthesis and the commercially-available powered knee prosthesis on two different testing days. On each day, their speed was held constant as they completed 3 replicate trials of each visual feedback target level and a no-feedback condition (total 12 trials). The blue dot in the center of the display, representing the stance time of the amputated-side (in seconds) averaged across the previous 5 strides, updated after every stride by moving up and down the y-axis

We created custom code for the real-time visual feedback display using Vicon DataStream SDK (VICON; Oxford, UK) and MATLAB (The MathWorks, Inc.; Natick, Massachusetts, USA) and displayed it on a 0.6-m computer monitor at eye-level, 1 m in front of the treadmill (Fig. 1). We calculated prosthetic-limb stance time as the amount of time we detected a vertical ground reaction force greater than 10% of the subject’s body weight, averaged it over the previous five strides [25] to reduce subjects’ tendency to generate large stride-to-stride corrections, and updated it after each ipsilateral toe-off event (Fig. 1, blue dot). The subject’s preferred stance time on each limb was extracted during the first trial, a no-feedback (None) trial, and used to set three visual feedback targets. Level 1 (L1) corresponded with the typical stance time of the prosthetic-limb, Level 3 (L3) corresponded with the stance time of the intact-limb, and Level 2 (L2) was set at the midpoint between L1 and L3. The targets remained constant to encourage consistency in walking patterns and were centered on the screen to blind the subject from which level they were targeting (Fig. 1). The display range remained +/− 0.2 s from the target line to maintain subjects’ perceived accuracy.

### Experimental design

Subjects visited the lab for a total of 3 days for powered knee prosthesis fitting and training with the prosthetist, testing with the prescribed (i.e. passive) knee prosthesis, and testing with the powered knee prosthesis. Each visit typically lasted 3 h. Subjects performed the prescribed prosthesis testing first to ensure familiarity with the protocol prior to introducing a new device. On the fitting and training day, the prosthetist aligned and tuned the control parameters of the powered prosthesis (Table 2), and subjects practiced walking with the powered prosthesis until they felt comfortable on the treadmill without assistance.

At the beginning of each testing day, we determined the subject’s self-selected walking speed on the treadmill (Table 1) and familiarized him/her with the Perceived Difficulty scale and visual feedback paradigm. To determine the self-selected speed, we started at a comfortable walking speed, increased/decreased the speed by 0.1 m/s increments until the subject stated that the speed was too fast/slow, repeated this increase/decrease procedure three times in each direction while recording the speed that was noted as too fast/slow, and averaged these six speeds for their final self-selected speed (modified from [26]). Prior to testing, we provided time for subjects to practice using the visual feedback, but we kept this time minimal (until the subject confirmed they were comfortable, usually 30 s) to prevent fatigue.

During testing, subjects completed a total of twelve 1.5-min walking trials with two or more minutes of rest between trials. We randomized the four conditions (i.e. None, L1–3) within three testing periods to prevent training time or fatigue from confounding our results. The first trial was None, in order to determine L1–3.

We recorded the ground reaction forces of each limb from a dual-belt treadmill (1000 Hz; Bertec Corp.; Columbus, OH, USA) and full-body motion using a 12-camera motion capture system (100 Hz; VICON; Oxford, UK). After each trial, we recorded subjects’ Perceived Difficulty using a 9-item Likert scale: 1 corresponded to “very easy”, and 9 corresponded to “very difficult”.

To evaluate amputees’ ability and strategy to modulate their walking pattern, we quantified their target-hitting accuracy and temporal gait measures (i.e. stance time, swing time, stance time asymmetry). To evaluate the associated effects on subjects’ locomotor function and proximal joints, we quantified peak anterior propulsive force (and asymmetry), posterior braking force, and hip joint power of each limb [27]. Due to the large variability between amputee subjects, we felt the need to use primarily global metrics (interlimb symmetry and perceived difficulty), and the hip is the only intact joint with which we can observe interlimb symmetry.

### Data processing

We selected nine consecutive strides from each trial, as this was the maximum number of consecutive strides in which subjects did not scuff the treadmill or touch the handrails across trials. For subject 5 only, we were unable to extract nine clean, consecutive strides from three trials (i.e. two None trials and one L1 trial), so we excluded these trials. For the visual feedback trials, we selected consecutive strides in which the subject was most consistently hitting the target. We low-pass filtered (Butterworth, 4th order, 7.5 Hz) the ground reaction force and motion data, baseline-corrected the ground reaction force data, and used a 20 N threshold to identify gait events. We calculated sagittal-plane hip joint kinetics after manually adjusting the inertial properties of the prosthesis segments (Visual 3D; C-Motion, Inc.; Germantown, MD, USA). We normalized ground reaction forces and hip power to body mass and identified peak braking/propulsive forces from the anterior-posterior ground reaction force. For peak hip power, we identified maximum hip power during the second power generating interval of the stance phase, as it aligns with anterior propulsion. To evaluate inter-limb symmetry, we used a standard asymmetry index (ASI) [7, 28]:

$$ ASI=\frac{x_i-{x}_p}{\left({x}_i+{x}_p\right)\ast 0.5}\ast 100, $$

where *x*_*i*_ and *x*_*p*_ are the outcome measure for each stride of the intact and prosthesis side, respectively. Target-hitting accuracy was calculated as the absolute value of the error between subjects’ prosthesis-side stance time and the target value for each stride using the same 10% body weight threshold.

### Statistical tests

We averaged the 3 replicate trials for each testing condition and used repeated-measures, two-way ANOVA (alpha = 0.05) to test for significant effects of the feedback and device (i.e. knee prosthesis type) factors (JMP; SAS Institute; Cary, NC, USA). The analysis included random subject effects and their interactions with feedback and device, and fixed effects for testing period. The model R-squared value exceeded 0.90 for our main response variables. We used the Shapiro-Wilk normality test (*p* < 0.01) to detect outlier trials (Table 3). When we found a significant main effect, we used Tukey’s honestly significant difference test (alpha = 0.05) to test for statistical difference between conditions. To investigate subject/device variance, we included within-subject comparisons for the kinetic measures (alpha = 0.05) (Fig. 4). We reported effect sizes as ${\eta}_p^2$ (SS_effect_/(SS_effect_ + SS_effect*subject_)), statistical power as 1-β, and mean and standard deviations throughout. Subject 5 did not have a passive prosthesis, so we did not include her in the statistical analysis to maintain equal sample sizes.

**Table 3:** ANOVA results with the inclusion/exclusion of each identified outlier trial

Response Measure	Included/excluded outlier trial	Device main effect	Feedback main effect	Interaction effect
Perceived difficulty	*Included*	0.157	**<0.001**	0.639
	*Excluded*	0.300	**<0.001**	0.507
Prosthesis-side peak braking force	*Included*	0.541	0.272	0.866
	*Excluded*	0.607	0.252	0.839
Intact-side peak hip power	*Included*	0.864	0.080	0.997
	*Excluded*	0.840	0.074	0.979

In total, we identified 3 outlier trials in 3 response variables (i.e. perceived difficulty, prosthesis-side peak braking force, intact-side peak hip power). However, the inclusion/exclusion of these identified outlier trials did not affect the significance of our results (below), so we did not exclude any trials. Because these outlier trials were not outliers in any other response variable, the removal of identified outliers may remove valuable information from this study as a whole

## Results

Subjects’ accuracy in hitting each target did not differ significantly between device (i.e. knee prosthesis type) or feedback conditions, but perceived difficulty significantly increased with visual feedback level (Table 4). Subject 5 had an average accuracy of 0.08 s (6% of stride time) with the powered prosthesis, and her perceived difficulty followed the same trend with an average score of 2, 5, 5, and 6 (None to L3).

**Table 4:** Perceived difficulty, additional temporal measures, and braking force

	Prescribed Prosthesis				Powered Prosthesis				ANOVA Results^*^		
	None	Level 1	Level 2	Level 3	None	Level 1	Level 2	Level 3	Device Main Effect	Feedback Main Effect	Interaction Effect
Target-hitting accuracy (s)	-	0.07± 0.05	0.06± 0.05	0.06± 0.04	-	0.08± 0.08	0.07± 0.06	0.05± 0.03	0.833	0.624	0.352
Perceived Difficulty	1±1	3±2	3±1	4±1	2±2^c^	3±1^b^	4±1^ab^	5±1^a^	0.157	**<0.001**	0.639
Stride Time (s)											
Prosthesis side	1.3±0.1	1.3±0.01	1.4±0.1	1.4±0.1	1.4±0.1^b^	1.3±0.1^b^	1.4±0.1^ab^	1.5±0.1^a^	0.243	**0.003**	0.959
Intact side	1.3±0.1	1.3±0.01	1.4±0.1	1.4±0.2	1.4±0.1^b^	1.3±0.1^b^	1.4±0.1^ab^	1.5±0.1^a^	0.183	**0.003**	0.973
Normalized Stance Time (% stride)											
Prosthesis side	64±3	64±3	64±3	64±3	62±2	63±2	63±2	63±1	0.400	0.519	0.645
Intact side	71±2	71±1	70±1	68±1	72±2^a^	71±2^a^	71±2^ab^	69±2^b^	0.528	**0.005**	0.872
Normalized Swing Time (% stride)											
Prosthesis side	36±3	36±3	36±3	36±3	38±2	37±2	37±2	37±1	0.400	0.519	0.645
Intact side	29±2	29±1	30±1	32±1	28±2^b^	29±2^b^	30±2^ab^	31±2^a^	0.405	**0.007**	0.749
Peak Braking Force (W/kg)											
Prosthesis side	0.6±0.2	0.6±0.2	0.6±0.2	0.7±0.2	0.7±0.2	0.7±0.2	0.7±0.2	0.7±0.2	0.541	0.272	0.866
Intact side	1.0±0.3	0.9±0.3	1.0±0.3	1.0±0.3	1.2±0.2	1.1±0.2	1.2±0.2	1.3±0.2	0.397	0.056	0.732

^*^Conditions without the same superscript letter (a-c) are significantly different with Tukey’s multiple comparisons adjustment (alpha = 0.05). No interaction effects were significant in this study, so all superscripts correspond to the feedback main effect and are included in only the powered prosthesis columns for simplicity

**Fig. 2:**
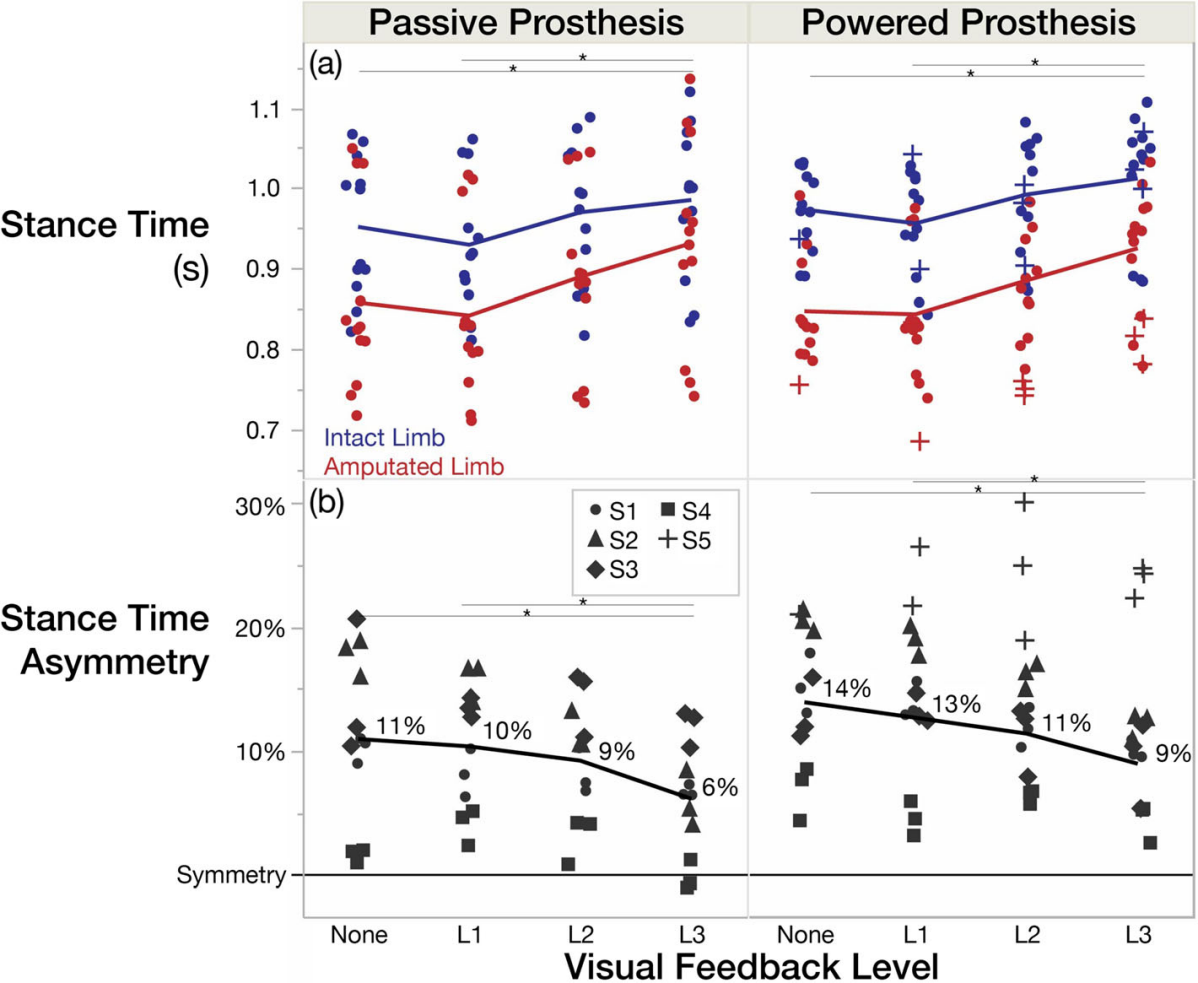
With visual feedback, stance time increased (to a lesser extent on the intact side), improving stance time symmetry. **a** Stance time in seconds shown for both devices. Blue corresponds to the intact limb, and red corresponds to the amputated limb. **b** Stance time asymmetry index with mean values labeled and subjects distinguished by point characters (S1–5). All point characters represent the mean of 9 strides from each trial, and the lines connect the means. Subject 5 was not included in the means, as she did not have a passive prosthesis

With increasing levels of visual feedback (i.e. None/L1 to L3), stance time on both limbs significantly increased (prosthesis-side *p* = 0.002, ${\eta}_p^2=$ 0.79; intact-side *p* = 0.005, ${\eta}_p^2=$ 0.74), and stance time symmetry significantly improved (*p* = 0.012, ${\eta}_p^2=$ 0.69) (Fig. 2). L3 significantly differed from None (and L1) conditions with 9% greater stance time on the prosthesis side, 4% greater stance time on the intact side, and 36% improved stance time symmetry with the powered prosthesis. Device main effects were not significant (prosthesis-side *p* = 0.856, 1-β = 0.96; intact-side *p* = 0.335, 1-β = 0.99; symmetry *p* = 0.179, 1-β = 0.90). Subject 5’s stance time increased by 7% on her powered prosthesis and 10% on her intact side, increasing her asymmetry by 13%.

**Fig. 3:**
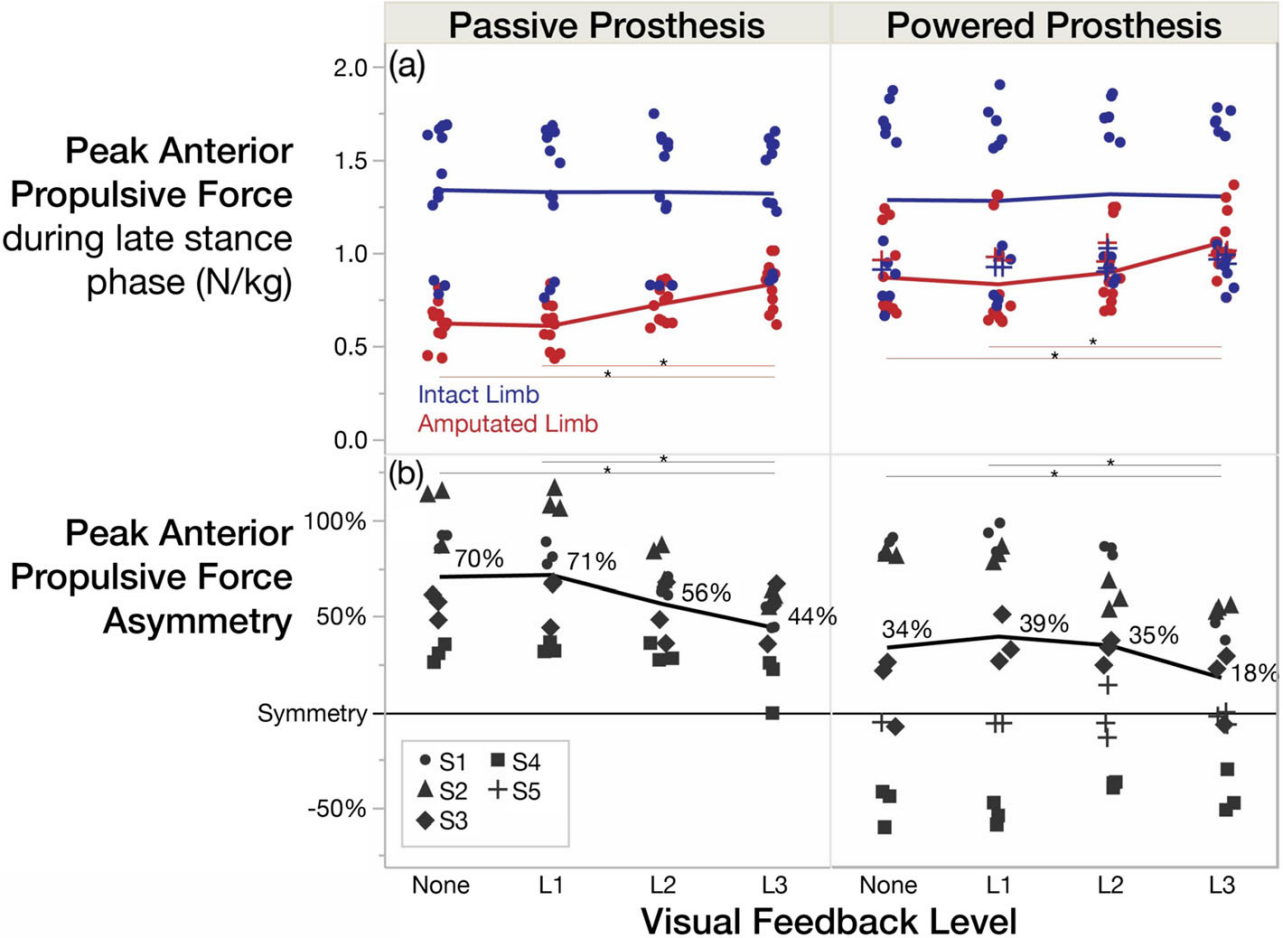
With visual feedback, subjects attained greater anterior propulsion from their amputated limb with the powered prosthesis, more closely matching the propulsion of the intact limb. **a** Peak anterior propulsion of each limb (normalized to body weight) shown for both devices. Blue corresponds to the intact limb, and red corresponds to the amputated limb. **b** Peak anterior propulsion asymmetry index with mean values labeled and subjects distinguished by point characters (S1–5). All point characters represent the mean of 9 strides from each trial, and the lines connect the means. Subject 5 was not included in the means, as she did not have a passive prosthesis

**Fig. 4:**
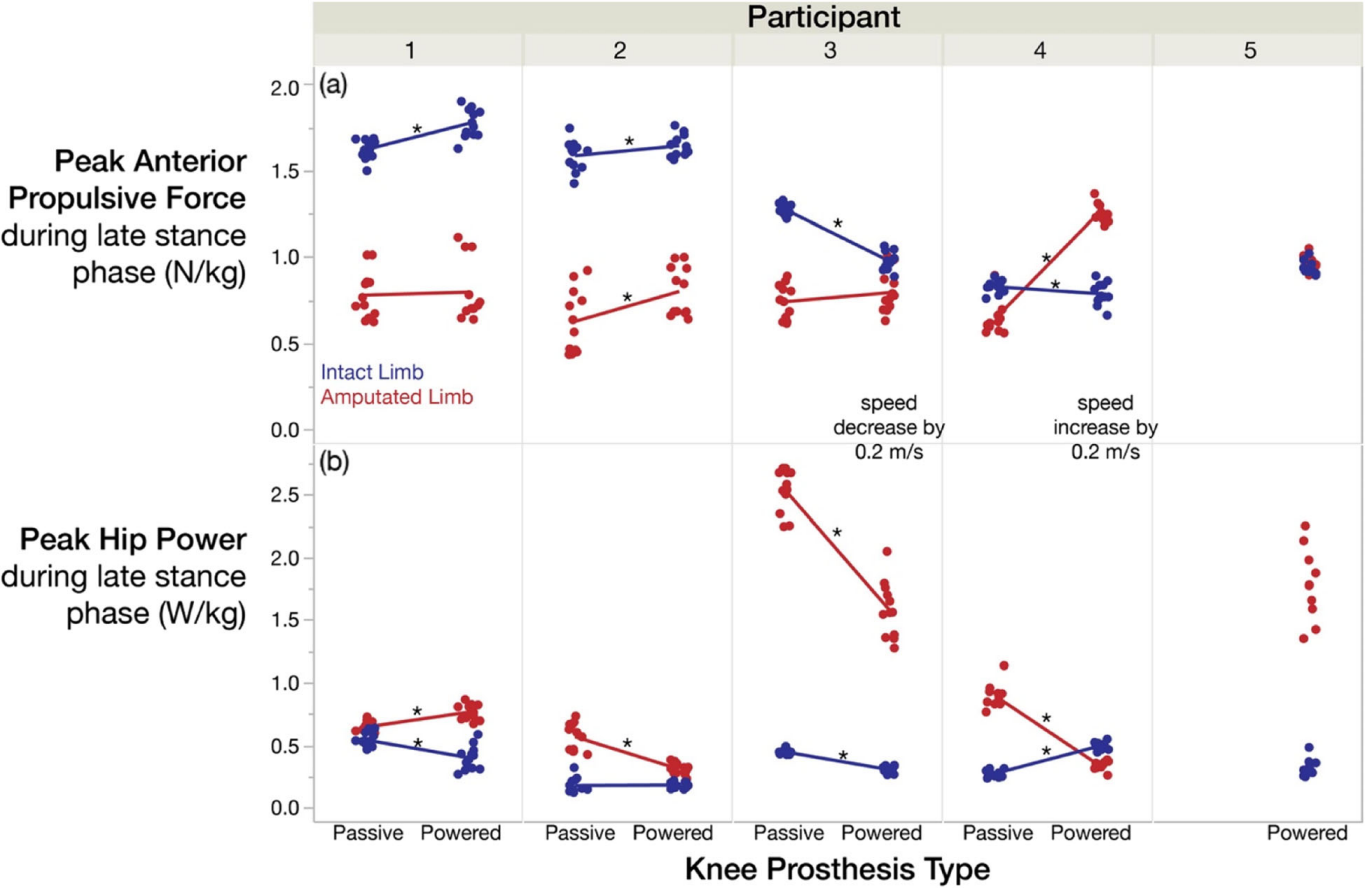
Subjects responded differently to each device, but generally exhibited similar or greater propulsion with the powered knee prosthesis, coinciding with reduced ipsilateral hip power that more closely matched their intact side. **a** Peak anterior-posterior ground reaction force and **b** peak hip power during late stance phase, normalized to body weight. All point characters represent the mean of 9 strides from each trial, and the lines connect the means

With increasing levels of visual feedback (i.e. None/L1 to L3), peak propulsive force significantly increased on the prosthesis-side only (prosthesis-side *p* = 0.012, ${\eta}_p^2=$ 0.66; intact-side *p* = 0.931, 1-β = 0.78), thus improving peak propulsion symmetry (*p* = 0.036, ${\eta}_p^2=$ 0.58) (Fig. 3). L3 significantly differed from None (and L1) with 22% increase in peak propulsion and 47% improvement in propulsion symmetry with the powered prosthesis. Device effects were not significant (prosthesis-side *p* = 0.128, 1-β = 0.56; intact-side *p* = 0.709, 1-β = 0.82; symmetry *p* = 0.146, 1-β = 0.81). Prosthesis-side peak propulsion averaged across conditions was 31% greater with the powered versus passive prosthesis (0.70 to 0.91 W/kg) and peak propulsion asymmetry was 48% less with the powered versus passive prosthesis (61 to 31%). Subject 5 was generally able to generate more propulsion from the powered prosthesis (and propulsion symmetry) than most other subjects (Fig. 4). Comparing L3 to None, her peak propulsion increased by 4% on the prosthesis side (0.96 to 1.00 W/kg) and 6% on the intact side (0.91 to 0.96 W/kg), improving her propulsion asymmetry by 40% (− 6 to − 4%).

Peak positive hip power did not significantly change with visual feedback (prosthesis-side *p* = 0.879, 1-β = 0.40; intact-side *p* = 0.080, 1-β = 0.97) or device (prosthesis-side *p* = 0.169, 1-β = 0.26; intact-side *p* = 0.864, 1-β = 0.90). However, individual subject comparisons demonstrate that peak positive hip power on the prosthesis-side was significantly reduced (i.e. closer to that of the intact side) with the powered prosthesis for the same subjects that exhibited greater ipsilateral propulsion with the powered prosthesis (Fig. 4).

All feedback/device interaction effects were statistically insignificant, but subject/device interactions accounted for more than 76% of the total variance for kinetic measures (Fig. 4) and 59% for stance time.

## Discussion

To our knowledge, this study is the first to evaluate the effects of extended stance time on an amputees’ prosthetic limb via visual feedback with the goal of achieving greater functional benefits with a powered knee prosthesis. Our results partially support our primary hypothesis, as subjects exhibited significantly improved stance time symmetry and propulsion symmetry with visual feedback. Our remaining hypotheses were not supported, as subjects responded inconsistently to the powered knee prosthesis. Given these results, we support rehabilitation and engineering approaches aimed at improving amputees’ gait symmetry, but individual modifications to the device (e.g. control parameters) and/or intervention (e.g. additional physical therapist/verbal cueing) may be required.

Temporal consistency across devices is consistent with previous studies [20, 29], and complete symmetry was not attained in our cohort. This lack of symmetric gait is likely due to the design of our visual feedback and combined limitations of the prosthesis (e.g. lack of a powered ankle) and/or altered musculoskeletal structure post amputation. In line with previous studies, we also observed an improvement in propulsion symmetry with stance time symmetry [30], without significantly increasing ipsilateral braking forces [31]. These results suggest that prostheses are capable of providing more assistance for amputees during locomotion if amputees use visual feedback to consciously modify their gait behavior, and heavy powered ankle components (implemented in experimental powered knee-ankle prostheses) may not be necessary to increase propulsive forces from the prosthesis.

Subjects responded differently to each prosthesis, but propulsion from the prosthetic limb was either comparable to or greater with the powered prosthesis compared to the passive prosthesis (Fig. 4). A decrease in walking speed can yield a decrease in propulsion, but Subject 3’s propulsion only decreased on the intact side when switching to the powered prosthesis (and decreasing his speed), suggesting the powered prosthesis provided greater assistance than his passive prosthesis. Subject 4 (likely due to his high functional k-level) even generated greater propulsive force with the powered prosthesis compared to his intact limb, reversing his propulsion asymmetry in the opposite direction (Figs. 3 and 4). This phenomenon is quite atypical in the amputee population [7], and we believe it originates from a more posterior position of his prosthetic limb relative to his center of mass during stance phase (compared to his intact limb during stance phase). A physical therapist characterized his gait pattern as having a shortened prosthetic-side hip flexion at initial contact, a lengthened mid-stance and terminal-stance phase, and greater limb extension prior to his intact-limb’s heel strike compared to other subjects. This limb extension is behavior physical therapists work hard to achieve to promote greater propulsion. Subjects may further benefit from physical therapist guidance/verbal cueing in addition to this visual feedback to achieve a more optimal posture and symmetric levels of propulsion.

For Subjects 2–4, the additional propulsion with the powered prosthesis coincided with a reduction in ipsilateral hip power, which is likely an indication of a proximal-to-distal redistribution of power [32]. Subject 3’s prosthesis-side hip power reduction with the powered versus passive prosthesis may not be attributed to a decrease in speed alone, as the magnitude of power reduction was approximately twice as large as the power reduction expected in able-bodied people with the same decrease in speed [33]. Subject 4 favorably exhibited a decrease in prosthesis-side hip power with the powered prosthesis, despite an increase in speed. In a previous study, the advantages of a microprocessor knee prosthesis versus a mechanical knee prosthesis did not yield greater prosthetic-side propulsion [28], but instead, the active flexion of the powered knee prosthesis in this study may enable greater propulsion and a reduction in ipsilateral hip power for some subjects.

One of the limitations of the powered knee prosthesis is the lack of a powered ankle. The biological ankle is important for providing push-off power for symmetric gait [32, 34]. The lack of a powered ankle might limit the level of symmetry that can be achieved by the amputees. Though, adding another powered joint distally would considerably increase the weight of the prosthesis and likely the hip power needed to swing the prosthesis. Therefore, it would be an interesting study to determine whether a powered ankle is beneficial for transfemoral amputees.

All subjects were able to modify their prosthetic-limb stance time to reach the visual feedback target provided, but we observed bilateral temporal changes with our unilaterally-targeted feedback. Intact-limb stance time also increased, but to a lesser extent than the prosthetic limb (Fig. 2), corresponding to a significant decrease in intact-limb stance time when normalized to stride time (Table 4). While off-loading the intact limb is a favorable outcome, the lack of change in prosthetic-limb stance time normalized to stride time (Table 4) suggest amputees have a reduced set of compensatory strategies and are unable to attain a significant variety of movement (particularly with the prosthesis). An increase in absolute swing time and overall stride time seemed to be necessary consequences when increasing absolute stance time with feedback. Previous studies have demonstrated unilateral propulsive biofeedback induces changes in the targeted limb only for both people with and without stroke [35, 36], but a prosthesis presents different challenges. Additionally, amputees may have less control over the prosthesis timing because the control parameters (related to swing flexion/extension timing) were fixed in this study. Physical therapy and/or adaptive powered prosthesis controllers may be necessary to increase amputees’ adaptability to locomotor strategies and gait symmetry.

Interestingly, subjects’ perceived difficulty increased with visual feedback level, but their target-hitting accuracy did not, suggesting they are capable of immediately walking more symmetrically, but they do not prefer it. Perceived difficulty with more symmetric gait may be associated with energetic requirements to maintain their lateral balance with longer single-support time on the prosthesis [13]. Long-term training has previously reduced transfemoral amputees’ energy expenditure [37], and may reduce subjects’ perceived difficulty with our visual feedback paradigm.

The improvement of both temporal and propulsive symmetry with visual feedback are very promising results for the use of this paradigm or similar therapies aimed at increasing amputees’ engagement with the prosthesis. As a next step, studying the effects of long-term training and varied dosage will be useful in determining the best way to implement this paradigm in the rehabilitation process [38]. Functional benefits may further increase over time, as demonstrated in a study of transtibial amputees after 3 weeks [39]. To further increase training time, this simple visual feedback paradigm may be modified for more cost-effective equipment for use at home (e.g. pressure-sensing treadmills), or even modified to use force-sensitive resistors in insoles and auditory cues for over-ground walking (e.g. [40]), though further research is required in this direction before implementation.

On the prosthetics engineering side, this feedback paradigm may be useful in designing powered prosthesis controllers that support more symmetric gait patterns (and increased engagement with the prosthesis) rather than fitting asymmetric gait patterns learned and reinforced with passive prostheses. For example, push-off timing has been shown to be critical in optimizing amputee users’ gait performance and metabolic cost [41], and researchers suggest powered knee extension timing may not be optimally timed for enhanced performance compared to passive knees [42]. The control parameters of a powered knee prosthesis have been shown to influence gait symmetry [17], but we have not yet developed a method of modifying the parameters to enhance gait symmetry. In our future work, we plan to develop a tuning policy for powered prostheses to maximize gait symmetry or other gait performance metrics. Moreover, the effects of maximizing user engagement with visual feedback in combination with control parameter tuning (e.g. automatic tuning [43, 44]) have yet to be investigated for improved amputee gait performance.

Perhaps most importantly, inter-subject differences, common among the amputee population [19, 20, 45] and suppressed in large group averages, are important to understand for improved prescription and personalized interventions. In addition, for evaluation purposes, it may be beneficial to develop and use more standardized and consolidated metrics of symmetry [46, 47] for a more comprehensive understanding of amputees’ quality of gait and comparison across studies. Note, for interventions such as visual feedback (and understanding specific behavioral changes as in this study), we support the use of targeted metrics, so the user is clear how to modify his/her gait pattern in response to the intervention.

### Study limitations

We acknowledge these preliminary results reflect the behavior of a small sample size, and may not represent the general clinical population, but small sample size is common for similar studies [48–50] and allows us to take a preliminary investigation into the individual differences that may arise. Latent effects associated with testing across multiple days and limited training time may confound our comparison between devices, so we recommend an acclimation time on the order of weeks (e.g. 3 months [51]) and repeated measures with each device for a more thorough comparison. Moreover, controlling foot prosthesis type may have reduced variability, but it would not be as clinically relevant and allow optimal performance with each knee prosthesis [51]. Previous studies comparing substantially different prosthetic feet did not find significant differences in anterior-posterior ground reaction forces or stance time [52, 53], so we believe our interventions predominantly contributed to the results in this study. Lastly, temporal and force asymmetry may be affected by walking speed [7], but we felt it was critical to have subjects walk at a comfortable speed to ensure maximal performance with each device. The comparisons between visual feedback levels (the primary factor being studied) are within the same walking speed.

## Conclusion

This study demonstrates the ability of amputee users to immediately manipulate their walking with a simple visual cue, thereby increasing their stance time and propulsive force symmetry with a commercially-available powered knee prosthesis. We believe this simple yet effective paradigm can be used as a rehabilitation tool to increase subjects’ use of their prosthesis during walking, but individual user differences should be considered before implementation. Using this type of feedback in combination with more advanced prosthesis control may allow amputee users to obtain greater functional benefits from powered prostheses. We encourage research on the effects of long-term use of simple and specific biofeedback metrics for amputee rehabilitation.

## Data Availability

The data from this study is available from the corresponding author upon reasonable request.

## Abbreviations

L1–3: Visual feedback levels 1–3
None: No visual feedback
